# Six-Week Endurance Exercise Alters Gut Metagenome That Is not Reflected in Systemic Metabolism in Over-weight Women

**DOI:** 10.3389/fmicb.2018.02323

**Published:** 2018-10-03

**Authors:** Eveliina Munukka, Juha P. Ahtiainen, Pere Puigbó, Sirpa Jalkanen, Katja Pahkala, Anniina Keskitalo, Urho M. Kujala, Sami Pietilä, Maija Hollmén, Laura Elo, Pentti Huovinen, Giuseppe D'Auria, Satu Pekkala

**Affiliations:** ^1^Institute of Biomedicine, University of Turku, Turku, Finland; ^2^Department of Clinical Microbiology and Immunology, Turku University Hospital, Turku, Finland; ^3^Faculty of Sport and Health Sciences, University of Jyväskylä, Jyväskylä, Finland; ^4^Department of Biology, University of Turku, Turku, Finland; ^5^Medicity Research Laboratory, University of Turku, Turku, Finland; ^6^Research Centre of Applied and Preventive Cardiovascular Medicine, University of Turku, Turku, Finland; ^7^Department of Health and Physical Activity, Paavo Nurmi Centre, University of Turku, Turku, Finland; ^8^Turku Centre for Biotechnology, University of Turku, Turku, Finland; ^9^Sequencing and Bioinformatics Service, Fundación para el Fomento de la Investigación Sanitaria y Biomédica de la Comunidad Valenciana (FISABIO-Salud Pública), Valencia, Spain

**Keywords:** exercise intervention, gut microbiota composition, gut microbiota function, systemic metabolites, cardiovascular effects

## Abstract

Recent studies suggest that exercise alters the gut microbiome. We determined whether six-weeks endurance exercise, without changing diet, affected the gut metagenome and systemic metabolites of overweight women. Previously sedentary overweight women (*n* = 19) underwent a six-weeks endurance exercise intervention, but two were excluded due to antibiotic therapy. The gut microbiota composition and functions were analyzed by 16S rRNA gene amplicon sequencing and metagenomics. Body composition was analyzed with DXA X-ray densitometer and serum metabolomics with NMR metabolomics. Total energy and energy-yielding nutrient intakes were analyzed from food records using Micro-Nutrica software. Serum clinical variables were determined with KONELAB instrument. Soluble Vascular Adhesion Protein 1 (VAP-1) was measured with ELISA and its' enzymatic activity as produced hydrogen peroxide. The exercise intervention was effective, as maximal power and maximum rate of oxygen consumption increased while android fat mass decreased. No changes in diet were observed. Metagenomic analysis revealed taxonomic shifts including an increase in *Akkermansia* and a decrease in *Proteobacteria*. These changes were independent of age, weight, fat % as well as energy and fiber intake. Training slightly increased Jaccard distance of genus level β-diversity. Training did not alter the enriched metagenomic pathways, which, according to Bray Curtis dissimilarity analysis, may have been due to that only half of the subjects' microbiomes responded considerably to exercise. Nevertheless, tranining decreased the abundance of several genes including those related to fructose and amino acid metabolism. These metagenomic changes, however, were not translated into major systemic metabolic changes as only two metabolites, phospholipids and cholesterol in large VLDL particles, decreased after exercise. Training also decreased the amine oxidase activity of pro-inflammatory VAP-1, whereas no changes in CRP were detected. All clinical blood variables were within normal range, yet exercise slightly increased glucose and decreased LDL and HDL. In conclusion, exercise training modified the gut microbiome without greatly affecting systemic metabolites or body composition. Based on our data and existing literature, we propose that especially *Akkermansia* and *Proteobacteria* are exercise-responsive taxa. Our results warrant the need for further studies in larger cohorts to determine whether exercise types other than endurance exercise also modify the gut metagenome.

## Introduction

The impact of the gut microbiota on human metabolic and immunologic health is increasingly recognized (Sekirov et al., 2010). Over a decade, a dysbiotic and metabolically unfavorable gut microbiota composition has been linked with several diseases (Ley et al., 2006; Cani et al., 2007; Munukka et al., 2012, 2014). Recently, animal models have shown that exercise impacts microbial abundance (Lambert et al., 2015; Welly et al., 2016), however, whether this holds true also in humans, is currently not well known as discrepant results have been published. To date, one study showed that exercise induced compositional changes in the human gut microbiota that were more pronounced in lean than in obese (Allen et al., 2017). Another study reported that combined eight-weeks aerobic and resistance exercise induced modest shifts in gut metagenomes despite no changes in the diversity of taxa or metabolic pathways (Cronin et al., 2018). Cross-sectional human studies have reported that professional rugby players diverge from body mass index (BMI)-matched non-athletes in several bacterial phyla (Clarke et al., 2014), and that normal weight active women differ from sedentary women in several taxa (Bressa et al., 2017). Nevertheless, the intake of gut microbiota -affecting nutrients including fiber was notably higher in athletes and active women than in non-athletes and sedentary, respectively, which may have influenced the outcomes. In addition, in the study of Bressa et al. the sedentary group had significantly higher body fat percentage and mass (Bressa et al., 2017), traits known to be highly associated with gut microbiota composition (Turnbaugh et al., 2009). Acute effects of exercise have also been studied using maximal bicycle ergometer test that showed an increase in the abundance of 7 out of 9 major taxa in patients with myalgic encephalomyelitis/chronic fatigue syndrome compared to 2 of 9 in healthy controls (Shukla et al., 2015). In addition, half marathon performed by amateurs was reported to affect gut microbiota composition, predicted functions and fecal metabolites (Zhao et al., 2018b).

Both exercise and gut microbiota are known to induce inflammation (Clark and Mach, 2017), which may be mediated by bacterial molecules-recognizing Toll-like receptors (TLR) (McKenzie et al., 2017). Thus, inflammation could be one link between the microbiota and the effects of exercise. Previously we have shown that the amine oxidase activity of Vascular Adhesion Protein 1 (VAP-1) is involved in a lipolysaccharide-induced inflammation model (Yu et al., 2006) and thus might potentially mediate the effects of the microbiota on inflammation. VAP-1 is a multifunctional protein with amine oxidase activity (Salmi and Jalkanen, 2017) that degrades primary amines and produces hydrogen peroxide, ammonium and aldehydes, which can modify the microenvironment to pro-inflammatory direction and cause detrimental effects on vasculature. Whether exercise regulates VAP-1 enzymatic activity is currently unknown.

In order to amplify the knowledge on the relationship between gut microbiota and exercise response in humans, this study determined the effects of six-weeks endurance exercise both on the gut microbiota composition and functions of overweight women. We hypothesized that exercise training would considerably change the microbiomes, which would be associated with both anti-inflammatory and inflammatory effects.

## Materials and methods

### Study subjects

Inclusion criteria were sedentary lifestyle and body mass index (BMI) >27.5 kg/m^2^. Exclusion criteria were major inflammatory gastrointestinal disorders (Crohn's disease, celiac disease, IBD), major eating disorders, diagnosed type 1 or 2 diabetes mellitus, cardiovascular diseases other than hypertension (previous stroke, transient ischemic attack, angina pectoris, myocardial infarction, use of medication for cardiovascular diseases other than hypertension, coronary bypass surgery, angioplasty or abnormal result on an exercise test indicating myocardial ischemia), hypothyroidism or other endocrine disease that may affect training or the study outcomes, and musculoskeletal diseases that could preclude the ability to perform training and testing. The subjects had not ingested antibiotics within 2 months prior to the sampling.

**Figure 1:**
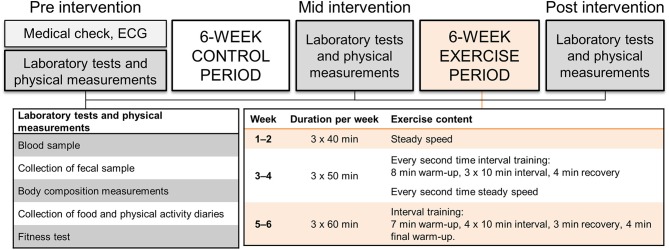
The study design, measurements and exercise protocol.

The study subjects were recruited through social media and by advertising the study in the newspaper of Central Finland area. Twenty-two women fulfilling the inclusion criteria were invited to the laboratory measurements based on phone interview. The medical history and current health status were evaluated *via* self-administered questionnaire and clinical examination by a physician including resting electrocardiogram. The physician confirmed that the subjects met the inclusion criteria. Two women were excluded: one due to the use of antidepressants that affect serotonin levels and one because of multiple allergies and anaphylactic reactions. Twenty women were thus included in the study. Afterwards, one dropped out and two were excluded of the microbiota analyses due to antibiotic therapy that is known to affect gut microbiota composition. The study design is presented in Figure 1. The study was conducted in accordance with the Helsinki Declaration and approved by the ethical committee of the Central Finland Health Care district (KSSHP) (KSSHP document number 2U/2015). A written informed consent was obtained from all study subjects prior to the study.

### Food diaries

The participants were advised to maintain their habitual *ad libitum* diet. The intake of total energy and energy-yielding nutrients were analyzed from 3-days food records (2 weekdays and 1 weekend day) using Micro-Nutrica software. The software has been continuously updated with new foods/drinks and recipes. Food records contained time of eating, foods/drinks consumed, and the amount of food/drink. Details of foods and drinks, i.e., type and commercial brand name, were entered in the records. Fourteen subjects completed the food diaries in all time points and were included in the analyses.

### Endurance exercise test

Incremental submaximal endurance exercise test was carried out using bicycle ergometer (SRM ErgoMeter, Schoberer Rad Messtechnik, Jülich, Germany). The intensity of 30 W was used at the beginning of the test, and was increased by 20 W every second minute until 85% of subject's age-predicted heart rate maximum (HR_max_) (Tanaka et al., 2001) was reached. The identical loading protocol was used in each test session and changes in gross efficiency and cycling economy was studied at 62 ± 3% of the estimated maximal power output at baseline (Pre). The subjects maintained a pedal cadence of 60 rpm that was observed continuously by a visual display on the cycle ergometer. Subjects perception of exertion was monitored using Borg's 6–20-point RPE scale (Borg, 1970) at the end of each stage. HR was monitored throughout the test (Polar S410; Polar Electro Oy, Kempele, Finland) and the average over the last 30 s of each stage was used for the subsequent analyses. To calculate estimated VO_2max_, the HR response was plotted against the workloads of each stage which were then extrapolated to the subject's estimated HR_max_ (Tanaka et al., 2001) to predict corresponding maximal power output. Predicted maximal oxygen uptake (VO_2max_) was calculated using the equation: VO_2max_ (ml kg^−1^ min ^−1^) = (1.8 × predicted maximal power output, kgm min^−1^)/body mass, kg) + 7. Oxygen uptake (VO_2_), carbon dioxide production (VCO_2_), and ventilation (VE) were determined continuously breath-by-breath using a metabolic cart (Oxycon Mobile, Jaeger, VIASYS Healthcare GmbH, Hoechberg, Germany). The average of the last 30 s of each stage was recorded for the analysis. The gas analyzer was calibrated on each testing day. Gross efficiency (%) for each stage was calculated dividing power output (W) by energy expended (J s^−1^) as previously described (Moseley and Jeukendrup, 2001). Energy expenditure was calculated as follows: [(3.869 × VO_2_) + (1.195 × VCO_2_)] × (4.186/60) × 1,000 (Moseley and Jeukendrup, 2001). In the calculation of energy expended, 1 J/s = 1 W. Blood. samples for lactate concentration were taken from the fingertip before and every second minute during the incremental loading, as well as 2 min after the exercise test. Blood lactate concentrations were analyzed with Biosen C-line Clinic analyzer (EKF Diagnostic, Cardiff, UK). Individual aerobic and anaerobic thresholds for the determination of training intensities were assessed using deflection points obtained by plotting the curves of blood lactate concentrations, ventilation, oxygen consumption and carbon dioxide production (Aunola and Rusko, 1986).

### Maximal isometric force

An electromechanical dynamometer (modified DAVID F 200 apparatus, David Health Solutions Ltd., Helsinki, Finland) was used to measure maximal voluntary isometric force of the bilateral knee extension action at a knee angle of 107°. A minimum of three trials was completed for each subject, and the best performance trial (kg) was used for the subsequent statistical analysis.

### Muscle thickness of *M. vastus lateralis*

The muscle thickness of *M. vastus lateralis* from the midpoint of proximal border of the patella and trochanter major of the right leg was measured with a compound ultrasonography (Aloka ProSound SSD-5500, Tokyo, Japan). At each ultrasonography measurement, three consecutive measures were taken and two closest values were averaged for further analyses.

### Endurance training

Subjects were asked to maintain individual habitual physical activity throughout the study. All prescribed training in the study was consistently supervised by qualified instructors. The training was designed to reflect a program aimed for physically active populations according to recommendations outlined by the American College of Sports Medicine. The intensity of the endurance training during the 6-weeks training period was controlled by HR (Polar S410; Polar Electro Oy, Kempele, Finland) associated with subject's individual aerobic and anaerobic threshold determined during the measurement before the training period. Subjects were instructed to maintain a constant pedaling frequency of ~60 rpm during each training session (controlled by metronome), while the magnetic resistance of the ergometer (Precor Studio Team Bike, Gym Equipment Ireland, Dublin, Ireland) was adjusted to achieve the required exercise intensity. Three training sessions were performed per week and carried out in groups of 2–4 subjects. During weeks 1–2, 40 min steady-state cycling of low intensity (below the aerobic threshold) was performed. During weeks 3–4, the duration of the exercise session was 50 min. Every other training session included three 10-min intervals of moderate intensity cycling (between the aerobic and anaerobic threshold) while rest of the training session was performed with low intensity cycling. Every other training session included only low intensity cycling. During weeks 5–6, the duration of training sessions were 60 min and included four 10-min intervals of moderate intensity cycling while rest of the training session was performed with low intensity cycling. The training intensity was verified by RPE scale and blood lactate measures in the beginning of the training period.

### Body composition measurement

Body height (cm) was measured by using a wall-fixed measuring device and weight (kg) determined with electronic scale. BMI was calculated as weight (kg)/height (m)^2^. Waist circumference was measured twice with a tape measure, and the mean value was used. Body composition, i.e., total body fat mass (FM, kg), FM percentage (FM%), android and gynoid FM and % and lean tissue mass (LM, kg), was assessed with dual-energy X-ray absorptiometry (DXA, Prodigy; GE Lunar Corp., Madison, WI, USA).

### Fecal sample collection, 16s rRNA gene sequencing and metagenome analyses

Subjects self-collected the fecal samples before the control period, and after the control period and the exercise training period. The samples were always collected over 72 h after the last exercise bout. Samples were frozen immediately at −20°C after collection, brought to laboratory frozen and stored at −80°C until processing. Total bacterial DNA was extracted using GTX stool kit and semi-automated GenoXtract machine (Hain Lifescience, Nehren, Germany) within 2 days after arrival. The gut microbiota composition of the samples from the baseline, before the exercise period and after the exercise period was analyzed by 16S rRNA gene amplicon sequencing. Variable region V4 of the bacterial 16S rRNA gene was amplified as described previously (Rintala et al., 2018), and the 16S rRNA gene libraries were sequenced with 2 × 250 bp paired-end reads on Illumina MiSeq system (Illumina, Inc.) using MiSeq v3 reagent kit (Illumina, Inc.). The sequences have been deposited to ENA with the accession number PRJEB27978.

The samples before and after the exercise training were further subject to metagenome analysis. DNA libraries were generated following Nextera XT Illumina protocol (#FC-131-1024, Illumina, San Diego, CA, USA) and 0.2 ng/ul of purified gDNA. The multiplexing step was performed using Nextera XT Index Kit (#FC-131-1096). The libraries were sequenced using 2 × 300 pb paired-end run (#MiSeq Reagent kit v3 #MS-102-3001) on a MiSeq Sequencer according to manufacturer's instructions (Illumina). After sequencing, quality assessment of the obtained reads was performed using “prinseq-lite” program (Schmieder and Edwards, 2011) applying following parameters: min_length: 50; trim_qual_right: 20; trim_qual_type: mean; trim_qual_window: 20. Paired-end fastq files resulting from Illumina sequencing were joined using “fastq-join” from “ea-tools” suite (https://expressionanalysis.github.io/ea-utils/, last web-site access 19/10/2017). Sequences passing quality controls were screened for ribosomal genes, i.e., Small Subunit (SSU) and Large Subunit (LS): 16S and 23S rRNA genes, respectively, using “sortmerna” program (Kopylova et al., 2012). Reads containing ribosomal gene fragments were passed to taxonomic analysis, and taxonomic annotation of SSU and LSU reads was carried out with SILVA Incremental Aligner (SINA) v1.2.10 using SILVA Release 123.1 (Pruesse et al., 2007, 2012). The rest of the reads were used for ORFs. The metagenome taxonomic data has been deposited to ENA with the accession number PRJEB27914.

The database of Clusters of Orthologous Groups (COGs) (Tatusov et al., 1997; Galperin et al., 2015) was used to identify the role of predicted proteins and to determine the relative abundance of broad functional categories. The latest version of the COGs database contains 4,631 orthologous proteins based on the annotation of 711 microbial genomes that represent the diversity of bacteria and archaea. All predicted proteins from the fecal samples were mapped onto the COGs database *via* BLASTP searches using a cut-off of 10^−10^ and selection of the best blast hit. The functional annotation of all ORFs was performed in two steps: (1) a BLASTP search using an e-value cut-off of 10^−10^ to filter out random matches and (2) selection of only one matching sequence based on the best blast hit to prevent cross-reference among genes. The gene abundances, the gene abundance enrichment in KEGG pathways, Principal component analysis (PCA) and Bray Curtis dissimilarity analysis were further performed using Microbiome Analyst (www.microbiomeanalyst.ca). After filtering genes with the lowest abundance (<2 hits), the total number of genes was 256 of which 132 were mapped to Microbiome Analyst database. The abundance range of the genes was 68-3773 before exercise and 47-3688 after exercise. When assessing the metagenome data the samples of two subjects clustered differently and were removed from the final analyses. Therefore, 15 subjects were included in the metagenome analyses.

### Blood samples, analysis of clinical variables and metabolomics

Blood samples were taken after overnight fasting over 72 h after the last exercise bout. The analysis of clinical variables (glucose, LDL and HDL cholesterol, free fatty acids, triglycerides and insulin) were analyzed with KONELAB 20XTi analyser (Diagnostic Products Corporation, Los Angeles, CA, USA). Homeostasis model assessment of insulin resistance (HOMA-IR) was calculated fasting glucose × fasting insulin/22.5.

Metabolic biomarkers were quantified from plasma using targeted high-throughput proton NMR metabolomics platform (Brainshake Ltd, Helsinki, Finland). This method provides simultaneous quantification of routine lipids, lipoprotein subclass profiling with lipid concentrations within 14 subclasses, fatty acid composition, abundant proteins and various low-molecular metabolites including amino acids, ketone bodies and gluconeogenesis-related metabolites in molar concentration units. The 14 lipoprotein subclass sizes were defined as follows: extremely large VLDL with particle diameters from 75 nm upwards and a possible contribution of chylomicrons, five VLDL subclasses (average particle diameters of 64.0, 53.6, 44.5, 36.8, and 31.3 nm), IDL (28.6 nm), three LDL subclasses (25.5, 23.0, and 18.7 nm), and four HDL subclasses (14.3, 12.1, 10.9, and 8.7 nm). The following components of the lipoprotein subclasses were quantified: phospholipids, triglycerides, cholesterol, free cholesterol and cholesteryl esters. The mean size for VLDL, LDL, and HDL particles was calculated by weighing the corresponding subclass diameters with their particle concentrations. Representative coefficients of variations over thousands of samples for the NMR-based metabolic measures have been reported before (Kettunen et al., 2016).

### VAP-1 concentration and enzymatic activity

Soluble VAP-1 (sVAP-1) levels were measured with an in-house sandwich ELISA as previously described (Aalto et al., 2014) and the enzymatic activity of VAP-1 as production of hydrogen peroxide as previously described (Aalto et al., 2012). Briefly, 96-well microtiter plates (MaxiSorp, Nunc, Roskilde, Denmark) were coated with a monoclonal anti-VAP-1 antibody overnight. Serum samples (diluted in 1:100) were added, and after incubation and subsequent washes bound VAP-1 was detected with another biotinylated anti-VAP-1 monoclonal antibody and streptavidin-horseradish peroxidase conjugate (GEHealthcare, Buckinghamshire, United Kingdom). Finally, BM Chemiluminescence ELISA substrate (Roche Diagnostics, Mannheim, Germany) was added, and the luminescence was measured with Tecan Infinite M200 plate reader (Tecan Group Ltd, Männedorf, Switzerland). The sVAP-1 values are presented as nanograms of protein in a milliliter of serum.

VAP-1 amine oxidase activity was assayed radiochemically using [7-14C]-benzylamine hydrochloride (spec. act. 57 mCi/mmol, Amersham, Little Chalfont, UK) as a substrate. The catalytic reaction was stopped by 100 μl of 2 mol/L citric acid, and aldehyde reaction products were extracted from the analyzed mixture into toluene containing 0.35 g/L diphenyloxazole. The amount of 14C-labeled benzaldehyde was quantified by scintillation counting with a β-counter (Wallac, Waltham, MA, USA). The activity of the enzyme was expressed as nanomoles of benzaldehyde oxidized by 1 mg lysate protein per hour. Protein concentrations were determined with Pierce bicinchoninic acid assay (Thermo Fischer, Waltham, MA, USA).

### Real-time quantitative PCR from whole blood

Total RNA was extracted of 500 μl of whole blood using Tri reagent (Fischer Scientific, Hampton, NH, USA) according to the supplier's protocol. Total RNA was reversely transcribed according to the manufacturer's instructions using High Capacity cDNA Synthesis Kit (Applied Biosystems, Foster City, CA, USA). Real-time PCR analyses were performed according to MIQE guidelines using in-house designed primers (Invitrogen, Carlsbad, CA, USA), iQ SYBR Supermix, and CFX96™ Real-time PCR Detection System (Bio-Rad Laboratories, Richmond, CA, USA). The primer sequences were as follows; *TLR4*: Fwd5′AAGCCGAAAGGTGATTGTTG′3 and Rev5′CTGAGCAGGGTCTTCTCCAC′3; *TLR5*: Fwd5′TCAAACCCCTTCAGAGAATCCC′3 and Rev5′TTGGAGTTGAGGCTTAGTCCCC′3; *GAPDH*: Fwd5′CCACCCATGGCAAATTCC′3 and Rev5′TGGGATTTCCATTGATGACAA′3. Each sample was analyzed in duplicate, and PCR cycle parameters were as follows: +95°C for 10 min, 40 cycles at +95°C for 10 s, at +56°C (*TLR4*) or +59°C (*TLR5*) or +60°C (*GAPDH*) for 30 s, and at +72°C for 30 s, followed finally by 5 s at +65°C. The relative expression levels of *TLR4* and *TLR5* were determined with CFX96™ Manager Software against the standard curve. Finally, the relative expression levels were normalized to the levels of the *GAPDH* housekeeping gene.

### Statistics

Statistical analyses were made using IBM SPSS Statistics 22 (Armonk, NY, USA). Shapiro Wilk's test in SPSS was used to assess the normality of distribution. The differences in normally distributed variables: body composition, metabolites, physical performance, energy intakes, and metagenome functions between different time points were studied with repeated measures analysis of variance (ANOVA) using the GLM procedure and Bonferroni *post-hoc* test. From the 16S rRNA gene amplicon sequencing and metagenome sequencing the group comparisons of gut microbiota were analyzed with non-parametric Wilcoxon matched-pair signed rank test and corrected for multiple comparison with Benjamini-Hochberg procedure. Regarding the taxonomic data, all analyses were made with QIIME from the randomly subsampled OTU table with rarefaction level matching the sample with the lowest total OTU count. The bacterial diversity of the samples (α-diversity metrics) and statistically significant differences in the OTU abundances were computed with QIIME. Taxonomic levels Phylum, Family and Genus were studied. The correlations between the non-normally distributed gut microbiota and other variables were determined using Spearman's rank correlation coefficient in SPSS. General linear model in SPSS was used to determine whether the changes in taxa occurred in response to exercise or whether they were dependent on age, weight, body fat %, android fat %, intake of total energy intake, sucrose or fiber.

## Results

### Exercise training significantly increased maximal power and oxygen uptake capacity and decreased blood lactate

Previously sedentary overweight women (*n* = 19) underwent the six-weeks guided endurance exercise intervention but two were excluded of the final analyses due to antibiotic therapy during the intervention. They had no intestinal or other chronic diseases or medications that could have affected the gut microbiota. While no changes in the physical performance were observed after the control period, both the maximal power and maximum rate of oxygen consumption (VO_2_max) increased after the exercise training period (Table 1). Blood lactate levels at submaximal workload decreased following the training period (Table 1) indicating improved aerobic metabolism.

**Table 1:** The physical performance before (Pre) and after the 6-weeks non-training control period (Mid) and after the 6-weeks endurance exercise training period (Post) (*n* = 17).

**Variable**	**Pre mean ±SD**	**Mid mean ±SD**	**Post mean ±SD**	***P*-value**
**ESTIMATED MAXIMAL VALUES BY SUBMAXIMAL ENDURANCE EXERCISE TEST**				
VO_2max_ (ml·min^−1^·kg^−1^)	29.8 ± 5.2	29.5 ± 5.6	32.5 ± 5.4^***^^###^	<0.001
Power (W)	182.9 ± 36.0	179.5 ± 38.5	202.6 ± 36.4^***^^##^	<0.001
**SUB-MAXIMAL VALUES AT** ~**60% OF ESTIMATED MAXIMUM POWER AT PRE**				
Heart rate (bpm)	147.1 ± 6.2	145.3 ± 10.0	138.8 ± 9.8^**^^##^	<0.001
Blood lactate (mmol·L^−1^)	3.0 ± 0.8	3.0 ± 0.8	2.3 ± 0.7^***^^###^	<0.001
VO_2_ (L·min^−1^) (*n* = 15)	1.45 ± 0.21	1.47 ± 0.24	1.45 ± 0.25	0.694
RER (*n* = 15)	1.09 ± 0.07	1.15 ± 0.08^#^	1.03 ± 0.06^***^^##^	<0.001
GE (%) (*n* = 15)	20.9 ± 1.2	20.8 ± 1.7	21.8 ± 1.9	0.054
**MUSCLE STRENGTH AND SIZE**				
MIF (kg)	154.4 ± 27.5	154.1 ± 29.9	147.8 ± 31.1	0.097
VLT (cm)	2.34 ± 0.42	2.39 ± 0.41	2.45 ± 0.41^#^	0.023

VO_2max_, maximal oxygen uptake; VO_2_, oxygen uptake; RER, respiratory exchange ratio; GE, gross efficiency; MIF, maximal isometric force of knee extensors; VLT, thickness of vastus lateralis muscle. Statistically significant change (

# *p < 0.05*,

## p < 0.01,

### p < 0.001) from the baseline (Pre);^*^Statistically significant change (

** p < 0.01,

*** *p < 0.001) from the post-control period (Mid)*.

### Exercise training did not lead to a significant weight loss but decreased android fat mass

Due to that the gut microbiota composition is known to be highly associated with weight and body composition (Turnbaugh et al., 2009), the body composition of the study subjects was determined with DXA, BMI and weight. In the beginning of the study the study participants (36.8 ± 3.9 years old) weighed 90.1 ± 15.7 kg, after the control period 89.9 ± 15.0 kg and after exercise training 89.1 ± 15.2 kg. The BMI in the beginning of the study was 31.7 ± 4.4 kg/m^2^, after the control period 31.7 ± 4.1 kg/m^2^ and after exercise training 31.4 ± 4.1 kg/m^2^. No significant changes after the control or exercise training period were observed in weight, blood pressure, waist circumference, BMI, total or gynoid FM, total, android or gynoid fat%, or visceral fat (Table 2). After the exercise training period, a reduction of android fat mass (*p* = 0.005) was measured, which was not found after the control period (Table 2).

**Table 2:** Body composition, age and blood pressure before the control period (Pre), after the control/before exercise (Mid) and after the exercise period (Post).

**Variable**	**Pre (*n* = 17) mean ±SD**	**Mid (*n* = 17) mean ±SD**	**Post (*n* = 17 SD) mean ±SD**
Age (years)	36.8 ± 3.9	36.8 ± 3.9	36.8 ± 3.9
BP syst (mmHg)	130 ± 12	131 ± 12	132 ± 12
BP dias (mmHg)	81 ± 11	82 ± 9	80 ± 6
Height (cm)	168.2 ± 6.1	168.2 ± 6.0	168.1 ± 6.0
WC (cm)	98.7 ± 13.0	100.9 ± 11.7	99.3 ± 11.0
Weight (kg)	90.1 ± 15.7	89.9 ± 15.0	89.3 ± 15.6
BMI (kg/m^2^)	31.8 ± 4.4	31.7 ± 4.2	31.4 ± 4.1
Android FM (kg)	3.87 ± 1.42	3.84 ± 1.39	3.75 ± 1.39^**^
Gynoid FM (kg)	7.29 ± 1.63	7.18 ± 1.56	7.09 ± 1.57
Total FM (kg)	39.46 ± 10.87	39.10 ± 10.47	38.55 ± 10.59
Total LM (kg)	46.80 ± 5.31	46.94 ± 5.69	46.64 ± 5.45
Android fat%	52.2 ± 5.6	52.0 ± 5.8	51.8 ± 6.0
Gynoid fat%	50.2 ± 4.5	50.1 ± 4.9	49.9 ± 4.7
Total fat%	43.6 ± 5.4	43.4 ± 5.6	43.1 ± 5.6
Visceral fat area (cm^2^)	122.6 ± 22.6	123.1 ± 22.1	122.6 ± 22.7

BP, blood pressure; syst, systolic; dias, diastolic; WC, waist circumference; BMI, body mass index; FM, fat mass; LM, lean mass. Statistically significant change (

** *p < 0.01) from the post-control period (Mid)*.

### Exercise slightly affected clinical variables

While the determined clinical variables were within normal healthy range, exercise training slightly increased serum glucose and decreased HDL and LDL cholesterol levels (Table 3). Free fatty acids decreased and triglycerides and insulin increased after the control period.

**Table 3:** Clinical characteristics of the fastsing blood samples of the study subjects before the control period (Pre), after the control/before exercise (Mid) and after the exercise period (Post).

**Variable**	**Pre (*n* = 17) mean ±SD**	**Mid (*n* = 17) mean ±SD**	**Post (*n* = 17 SD) mean ±SD**
Glucose (mmol/L)	5.25 ± 0.30	5.15 ± 0.30	5.35 ± 0.31^**^
HDL (mmol/L)	1.30 ± 0.34	1.38 ± 0.34	1.13 ± 0.28^**^
LDL (mmol/L)	2.60 ± 0.59	2.72 ± 0.60	2.50 ± 0.56^**^
FFA (mmol/L)	699.29 ± 355.75	403.88 ± 195.87^##^	427.24 ± 127.94
Trigly (mmol/L)	0.95 ± 0.41	1.13 ± 0.65^#^	1.10 ± 0.51
Insulin (IU/L)	54.94 ± 28.82	77.80 ± 33.99^##^	77.32 ± 40.95
HOMA-IR	12.9 ± 6.8	18.1 ± 8.3	18.7 ± 10.6

FFA, free fatty acids; Trigly, triglycerides; HOMA-IR, homeostatic model assessment of insulin resistance (

# *p < 0.05*,

## p < 0.01, ^###^p < 0.001) from the baseline (Pre); Statistically significant change (

** *p < 0.01) from the post-control period (Mid)*.

### No changes in total energy intake, intake of energy-yielding or gut microbiota-affecting nutrients were observed after the exercise period

As dietary composition affects the gut microbiota, the total daily energy and the intake of energy-yielding nutrients, were analyzed from the 3-days food records. In addition, the energy % derived from the nutrients as well as daily intake of bread, other grain products, vegetables, fruits, berries, meat, fish, fermented milk products and cheeses were analyzed. Fourteen subjects completed the food diaries in all time points. No changes in their intake were observed after the control period or the exercise training period (Table 4). However, the energy % derived from starch slightly increased after the exercise period (*p* = 0.033).

**Table 4:** Total daily energy intake and intake of energy yielding nutrients before control period (Pre), after control/before exercise (Mid) and after exercise period (Post).

**Variable**	**Pre (*n* = 14) mean ±SD**	**Mid (*n* = 14) mean ±SD**	**Post (*n* = 14) mean ±SD**
Energy, kcal/day	*2, 122*±512	*1, 891*±609	*1, 975*±493
Protein, g/day	88.5 ± 34.7	92.1 ± 28.1	76.3 ± 21.7
Fat, g/day	88.4 ± 38.4	83.0 ± 75.2	75.2 ± 28.7
Carbohydrates, d/day	229.8 ± 62.6	217.2 ± 57.8	215.5 ± 70.5
Starch, g/day	104.6 ± 29.6	91.3 ± 22.4	104.5 ± 33.4
Sucrose, g/day	39.3 ± 19.9	34.3 ± 19.8	36.6 ± 21.7
Fiber, g/day	19.9 ± 5.0	19.3 ± 4.6	19.3 ± 7.3
Protein, E%	16.7 ± 3.8	18.4 ± 3.6	16.7 ± 2.7
Fat, E%	36.9 ± 9.8	36.5 ± 6.5	35.6 ± 5.5
Carbohydrate, E%	45.1 ± 11.5	43.8 ± 7.9	46.7 ± 5.3
Starch, E%	20.7 ± 6.3	19.2 ± 4.6	22.8 ± 3.4^*^
Sucrose, E%	7.4 ± 2.8	6.5 ± 3.1	7.5 ± 2.7
Fiber, g/MJ	2.3 ± 0.6	2.4 ± 0.7	2.5 ± 0.7
Bread, g/day	85.1 ± 32.4	84.0 ± 39.9	87.2 ± 57.6
Other grain products, g/day	130.1 ± 68.2	117.3 ± 50.0	134.7 ± 75.1
Vegetables, g/day	309.1 ± 133.6	306.6 ± 130.3	270.7 ± 127.5
Fruits and berries, g/day	197.3 ± 149.8	166.8 ± 114.8	182.6 ± 103.8
Fermented milk products g/day	144.4 ± 126.3	157.8 ± 108.8	97.1 ± 101.2
Cheeses, g/day	44.6 ± 27.5	39.2 ± 23.6	49.1 ± 26.8
Meat, g/day	143.6 ± 143.2	111.4 ± 65.8	82.0 ± 51.5
Fish, g/day	23.0 ± 42.0	31.1 ± 34.2	14.4 ± 21.1

Fourteen subjects completed the food diaries in all time points. kcal, kilocalories; g/day, grams per day; E%, energy percentage calculated as: 9.082 × (g/day divided by kcal/day) × 100 for fat, and as: 4.063 × (g/day divided by kcal/day) × 100 for protein, carbohydrate, starch, sucrose and fiber; g/MJ, grams per megaJoule. Statistically significant change (

* *p < 0.05) from the post-control period (Mid)*.

### Microbiome analysis revealed that exercise training modified the composition and functions of the gut microbiota

Due to that the exercise intervention was successful in increasing the maximal power and maximum rate of oxygen consumption, and no gross changes in weight, body composition or diet occurred after the exercise period, we were confident to detect the specific effects of exercise training on the gut microbiota if there were any.

**Figure 2:**
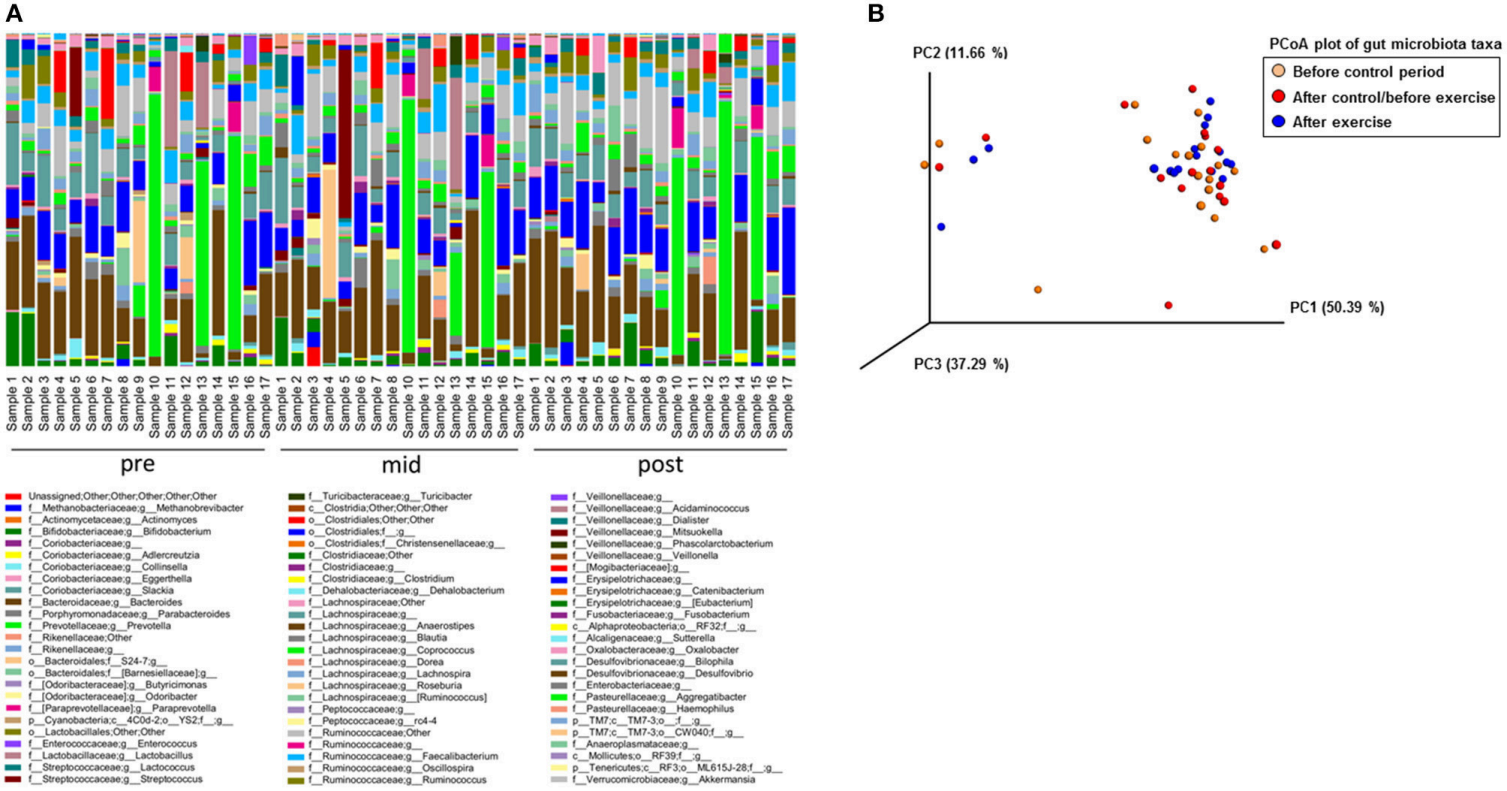
The 16S rRNA gene amplicon sequencing failed to show differences in the microbiota composition between the samples collected before the control period (Pre), after the control period/before exercise (Mid) and after the exercise (Post) period. **(A)** The genus level abundance analyzed with Illumina MiSeq 16S rRNA gene amplicon sequencing show great inter-individual variation that may have caused the lack of significant differences between the samples collected before the control period (Pre, *n* = 17), after the control period/before exercise (Mid, *n* = 17) and after the exercise period (Post, *n* = 17). **(B)** PCoA plot of the 16S rRNA gene amplicon sequenced samples before the control period (*n* = 17), after the control/before the exercise period (*n* = 17) and after the exercise period (*n* = 17) shows an important inter-individual variation in the exercise responsiveness.

With 16S rRNA gene amplicon sequencing, no governing effects on gut microbiota were observed after the control period, while after the exercise period the genus *Streptococcus* tended to decrease (*p* = 0.16). The read count in 16S rRNA gene sequencing was 49,761–207,043 per sample. The variation in the genus level composition of the gut microbiota between the subjects appears to be vast in all analyzed time points (Figure 2A). The Principal component analysis (PCoA) showed that the lack of abundant changes may have been due to relatively heterogeneous responses of the subjects to exercise training and inter-individual variation in gut microbiota (Figure 2B).

**Figure 3:**
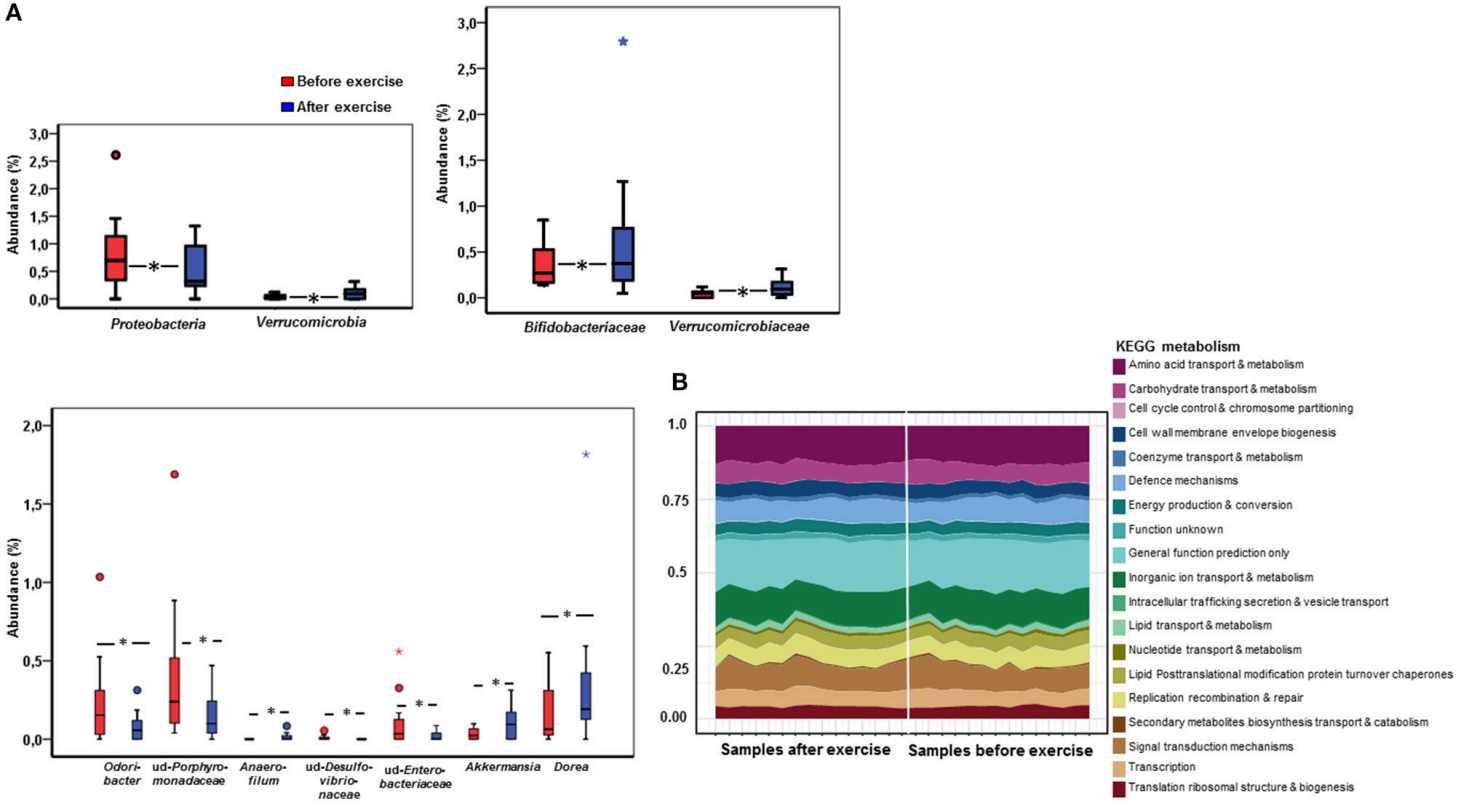
The fecal metagenome analysis reveals that exercise causes shifts in the microbiome at taxonomic while no changes in the major functional pathways were detected. **(A)** The box plots in the figure show phylum, family and genus level abundances before (*n* = 15) and after (*n* = 15) the exercise period of the significantly changing gut microbiota according to the metagenome analysis. An increase was detected in *Verrucomicrobia, Verrucomicrobiaceae, Bifidobacteriaceae, Akkermansia, Anaerofilum* and *Dorea* while *Proteobacteria, Odoribacter* and unidentified *Desulfovibrionaceae, Porphyromonadaceae* and *Enterobacteriaceae* decreased. **(B)** In the metagenomes no significant differences in the major KEGG metabolic pathways were detected between the samples before and after exercise according to the analysis performed with Microbiome Analyst. When assessing the metagenome data the samples of two subjects clustered differently and were removed from the final analyses. Therefore, 15 subjects were included in the metagenome analyses.

To obtain a deeper insight into the possible compositional and functional shifts of the gut microbiota, metagenome analysis was performed from the samples before and after the exercise period. The mean read count in the samples before exercise training was 579,673 ± 230,079, and after exercise training 583,651 ± 351,819. In metagenome analysis the samples of two subjects clustered differently and were excluded of the final analyses. In response to exercise training, no differences in gut microbiota α-diversity or the phylum level abundancies or the relationship of *Bacteroidetes* or *Firmicutes* were found (data not shown). However, the Jaccard distance of genus level β-diversity increased from 0.449 to 0.465 (*p* = 0.014) in response to exercise training, while phyla and family levels did not change. In addition, exercise training resulted in a decrease of gram-negative *Proteobacteria* (*p* = 0.039, Figure 3A) and an increase in *Verrucomicrobia* (*p* = 0.038). Specifically, exercise increased *Verrucomicrobiaceae* (*p* = 0.038) and *Bifidobacteriaceae* (*p* = 0.015; Figure 3A). Further, the genus *Dorea* (*p* = 0.028), *Anaerofilum* (*p* = 0.037) and *Akkermansia* (*p* = 0.038) increased while unidentified *Porphyromonadaceae* (*p* = 0.016), *Odoribacter* (*p* = 0.033), unidentified *Desulfovibrionaceae* (*p* = 0.037), and unidentified *Enterobacteriaceae* (*p* = 0.036) decreased after exercise training (Figure 3A).

We further determined using general linear model whether the changes in taxa occurred in response to exercise training or whether they were dependent on age, weight, body fat %, android fat %, intake of total energy intake, sucrose or fiber (Table 5). The changes in *Bifidobacteraceae*, and unidentified *Desulfovibrionaceae* and *Enterobacteriaceae* were dependent on all variables except age. The results suggest that only *Proteobacteria, Verrucomibrobia, Verrucomicrobiaceae*, and *Akkermansia* respond to exercise training independently of above-mentioned variables.

**Table 5:** The dependence of the taxonomic changes in response to exercise training on age, weight, body fat %, android fat %, intake of total energy intake, sucrose and fiber.

	**Mean** ±**SD**		***P*****-values**							
	**Before exercise**	**After exercise**	**Crude^a^**	**Adj Age**.	**Adj Weight**.	**Adj Fat %**.	**Adj Android fat %**.	**Adj Energy, E%**.	**Adj Fiber**.	**Adj Sucrose E%**.
**PHYLA LEVEL**										
*Proteobacteria*	1.05 (0.94)	0.65 (0.63)	**0.039**	**0.031**	**0.033**^b^ **0.034**^c^	**0.036**^b^ **0.032**^c^	**0.031**^b^ **0.026**^c^	**0.045**^b^ 0.078^c^	**0.038**^b^ **0.012**^c^	**0.043**^b^ 0.073^c^
*Verrucomicrobia*	0.03 (0.04)	0.10 (0.10)	**0.038**	**0.043**	**0.031**^b^ **0.037**^c^	**0.020**^b^ **0.012**^c^	**0.037**^b^ **0.045**^c^	**0.017**^b^ **0.013**^c^	0.054^b^ **0.021**^c^	**0.017**^b^ **0.017**^c^
**FAMILY LEVEL**										
*Bifidobacteriaceae*	0.41 (0.27)	1.18 (0.27)	**0.015**	0.198	0.179^b^ 0.199^c^	0.194^b^ 0.182^c^	0.184^b^ 0.179^c^	0.275^b^ 0.567^c^	0.356^b^ 0.385^c^	0.289^b^ 0.553^c^
*Verrucomicrobiaceae*	0.03 (0.04)	0.10 (0.10)	**0.038**	**0.043**	**0.031**^b^ **0.037**^c^	**0.020**^b^ **0.012**^c^	**0.037**^b^ **0.045**^c^	**0.017**^b^ **0.013**^c^	**0.017**^b^ **0.021**^c^	**0.017**^b^ **0.017**^c^
**GENUS LEVEL**										
*Akkermansia*	0.03 (0.04)	0.10 (0.10)	**0.038**	**0.038**	**0.028**^b^ **0.035**^c^	**0.018**^b^ **0.011**^c^	**0.033**^b^ **0.044**^c^	**0.015**^b^ **0.010**^c^	**0.048**^b^ **0.017**^c^	**0.014**^b^ **0.014**^c^
*Dorea*	0.16 (0.20)	0.35 (0.47)	**0.028**	0.099	0.054^b^ 0.095^c^	0.099^b^ 0.109^c^	**0.050**^b^ 0.080^c^	**0.009**^b^ 0.145^c^	**0.033**^b^ 0.064^c^	0.159^b^ 0.138^c^
*Odoribacter*	0.23 (0.29)	0.09 (0.10)	**0.033**	**0.049**	0.059^b^ **0.049**^c^	0.055^b^ **0.016**^c^	0.056^b^ **0.057**^c^	0.087^b^ **0.044**^c^	0.081^b^ **0.036**^c^	0.067^b^ 0.074^c^
*ud-Desulfovibrionaceae*	0.01 (0.02)	0.00 (0.00)	**0.037**	**0.038**	0.075^b^ 0.076^c^	0.076^b^ 0.071^c^	0.071^b^ 0.074^c^	0.123^b^ 0.174^c^	0.145^b^ 0.101^c^	0.109^b^ 0.199^c^
*ud-Enterobacteriaceae*	0.10 (0.17)	0.02 (0.03)	**0.036**	**0.027**	0.139^b^ 0.137^c^	0.091^b^ 0.137^c^	0.108^b^ 0.126^c^	0.276^b^ 0.290^c^	0.162^b^ 0.256^c^	0.253^b^ 0.283^c^
*ud-Porphyromonadaceae*	0.38 (0.46)	0.15 (0.16)	**0.016**	**0.045**	**0.045**^b^ **0.029**^c^	**0.038**^b^ **0.016**^c^	**0.044**^b^ **0.039**^c^	0.059^b^ 0.065^c^	0.079^b^ 0.084^c^	**0.048**^b^ 0.063^c^

a Crude p-value,

b *Adjusted (Adj.) for the value before exercise*,

c *Adjusted for change. The statistically significant p-values are highlighted in bold*.

**Figure 4:**
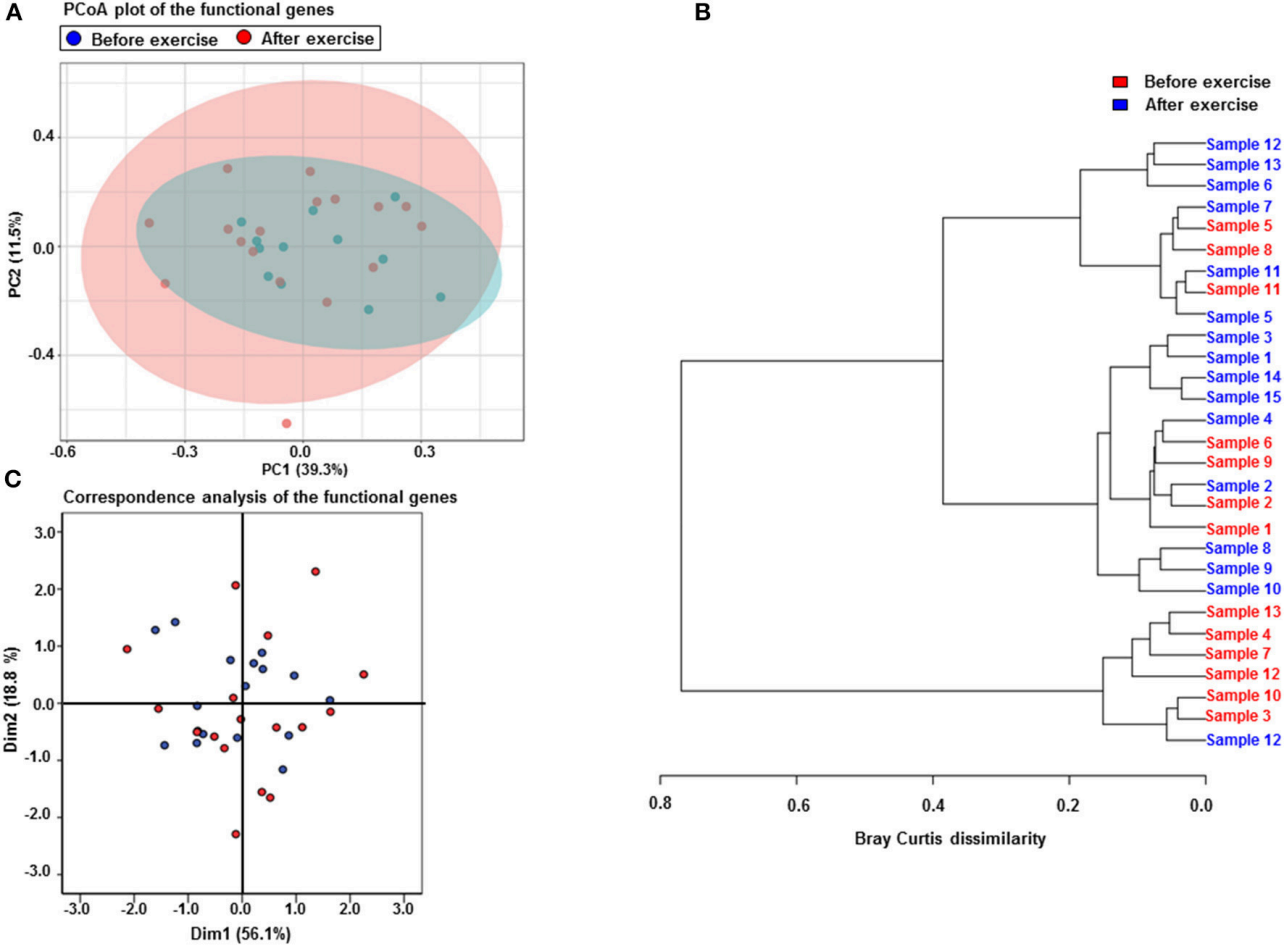
Exercise training increases the variability of the functional genes but only half of the subjects microbiomes respond to exercise. **(A)** PCoA plot of the functional genes shows that the samples before (*n* = 15) and after (*n* = 15) the exercise cluster closely together indicating their similarity. The first component explains 39.3% and the second 11.5% of the variability. **(B)** The Correspondence analysis of the functional genes shows that the samples before the exercise are less variable (more close to the centroid) than the samples after the exercise training indicating that the exercise has caused shifts in the gene abundances. **(C)** The Bray Curtis dissimilarity analysis shows that only approximately half of the subjects microbiomes have considerably responded to the exercise training, i.e., have Bray Curtis index close to 0.8.

The metagenomics assessment of the functional genes revealed no changes in the major pathways after the exercise period (Figure 3B). Accordingly the enrichment of pathways was not altered as shown in Table 6. The same pathways were similarly enriched in the study subject's microbiomes before and after the exercise training. Of the enriched pathways only Polyketide sugar unit biosynthesis remained significant after correcting for multiple comparisons (FDR). However, a closer analysis of individual genes showed that several gut microbiota functions decreased after the exercise period (Table 7). Despite finding post-intervention alterations in these genes, it was evident from the PCoA (Figure 4A) and Bray Curtis analyses that the samples before and after the exercise training cluster closely together indicating their similarity and that only approximately half of the subject's microbiome had considerably responded to the exercise training, i.e., had Bray Curtis index close to 0.8 (Figure 4B). In agreement, the Correspondence analysis shows that the samples before exercise are less variable (more close to the centroid) than the samples after exercise indicating that the exercise has caused shifts in COGs, which, nevertheless, are relatively heterogeneous (Figure 4C).

**Table 6:** Pathway enrichment before and after the exercise training.

**Pathway**	**Total genes**	**Expected**	**Hits**	***P*-value**	FDR^*^
Polyketide sugar unit biosynthesis	4	0.121	3	0.000103	0.0152
Streptomycin biosynthesis	12	0.362	3	0.00474	0.351
Selenocompound metabolism	15	0.452	3	0.00919	0.419
Amino sugar and nucleotide sugar metabolism	64	1.93	6	0.0113	0.419
Glycine, serine and threonine metabolism	78	2.35	6	0.0279	0.496
Thiamine metabolism	23	0.693	3	0.0301	0.496
Secondary bile acid biosynthesis	1	0.0301	1	0.0301	0.496
Biosynthesis of ansamycins	1	0.0301	1	0.0301	0.496
Biosynthesis of vancomycin group antibiotics	1	0.0301	1	0.0301	0.496

* *FDR, p-value corrected for multiple comparison with Benjamini-Hochberg procedure*.

**Table 7:** Changes in the abundance of the metabolic genes in response to the exercise training.

**Gene name**	**Function and mapping to KEGG pathway (pathway ID)**	**Change after exercise**	***F***	***P*-value**
7-keto-8-aminopelargonate synthetase or related enzyme	Biotin synthesis, ec00780	−73.197	5.106	0.047
FAD synthase	Riboflavin synthesis, ec00740	−45.091	7.474	0.023
Transcriptional regulators containing an AAA-type ATPase domain and a DNA-binding domain	ATPases Associated with diverse cellular Activities	−115.417	6.222	0.030
Aspartate ammonialyase	Alanine, Aspartate and Glutamate metabolism (ec00250)	−44.917	5.085	0.048
Cytochrome bd-type quinol oxidase, subunit 1	Terminal oxidase that produces a proton motive force, Oxidative phosphorylation (syne00190)	−36.379	5.238	0.043
K+-transporting ATPase, A chain	High affinity ATP-driven K+ transport system, Signal transduction (ko02020)	−43.356	5.130	0.047
Uncharacterized membrane protein YbjE, DUF340 family	DUF340 family includes lysine exporter LysO (YbjE)	−33.833	7.441	0.020
Transcriptional regulator of aromatic amino acids metabolism	Regulation of aromatic amino acids metabolism (map01230?)	−130.106	5.852	0.039
Transcriptional regulator of acetoin/glycerol metabolism	Activation of acetoin/glycerol metabolism	−136.894	5.130	0.047
Transcriptional regulator containing PAS, AAA-type ATPase, and DNA-binding Fis domains	Regulation of transcription by ATPase and DNA and protein or ligand binding	−167.189	6.203	0.030
ABC-type cobalamin transport system, permease component	B12 vitamin-derivative cobalamin transport system (cx02010)	−34.583	4.822	0.050
L-rhamnose isomerase	Fructose and mannose metabolism (ec00051)	−54.091	5.798	0.039
Uncharacterized protein YqfA, UPF0365 family	Predicted inner membrane oxidoreductase	−65.030	5.490	0.041

We further looked at the changes in body composition and clinical variables in those subjects whose microbiomes changed after exercise training according to Bray Curtis index. In this subgroup no significant changes in body composition occurred, and the change in android fat mass was no longer significant. Of the clinical variables the change in HDL, LDL and glucose remained significant while no other changes were detected.

### Association of the diet with metagenomic functions

As several reduced metagenomic functions were related to protein metabolism and B vitamin synthesis, we analyzed the associations between total protein, meat, vitamins B2 and B12 intake, the metagenomic functions and the abundance of microbial taxa. We found that aspartate ammonialyase after the exercise training associated inversely with protein intake before the exercise training (*r* = −0.720, *p* = 0.019) and B2 vitamin intake after the exercise training (*r* = −0.648, *p* = 0.043), and meat intake after the exercise training with the transcriptional regulator of aromatic amino acid metabolism (*r* = −0.774, *p* = 0.024).

### Exercise training had favorable cardiovascular effects

**Figure 5:**
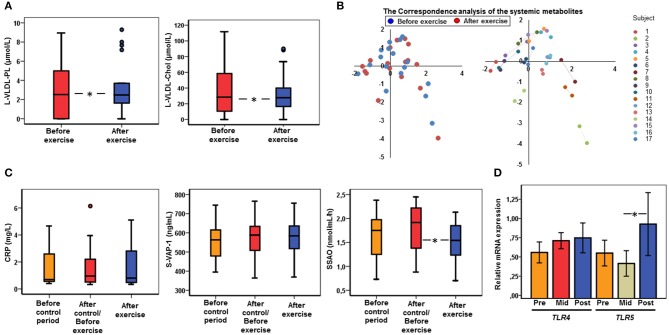
The cardiovascular and inflammatory changes in response to exercise. **(A)** After the exercise period (*n* = 17) the phospholipids (PL) and cholesterol (C) in large (L) VLDL particles were decreased compared to the samples before exercise (*n* = 17) as determined with NMR metabolomics. **(B)** The Correspondence analysis of the metabolites shows inter-individual variation in the metabolites and different clustering of the samples before (*n* = 17) and after (*n* = 17) the exercise (on left), and moreover, the samples of each subject before and after the exercise tend to cluster together (on right) indicating no gross changes in metabolites in response to exercise. **(C)** No differences in serum CRP levels were detected between the time points. After the exercise period (*n* = 17) VAP-1 enzyme activity (SSAO) decreased but no changes in VAP-1 protein concentration were observed. **(D)** After the exercise period (Post, *n* = 17) *TLR5* mRNA increased but no changes in *TLR4* were observed compared to the samples before the control period (Pre, *n* = 17) and after the control/before exercise (Mid, *n* = 17).

Exercise training decreased plasma phospholipids and cholesterol in large VLDL particles (*p* < 0.05 for both, Figure 5A) that, however, were not associated with gut microbiota. Somewhat surprisingly, exercise training did not significantly alter any other serum metabolites. The Correspondence analysis shows that it may be due to different clustering and inter-individual variation of samples before and after the exercise training (Figure 5B), and moreover, because the samples of each subject before and after exercise tended to cluster together indicating no gross changes in metabolites in response to exercise training (Figure 5B).

No changes were observed in serum CRP levels at any time point (Figure 5C). Exercise training decreased the enzymatic activity of VAP-1 (*p* = 0.011, Figure 5C), while such decrease was not seen in protein concentration, suggesting induction of an endogenous inhibitor. No significant differences in the expression levels of *TLR4* were found while *TLR5* expression significantly increased during the exercise period (*p* = 0.029, Figure 5D).

### The associations of gut microbiota with body composition disappear after the exercise period

**Figure 6:**
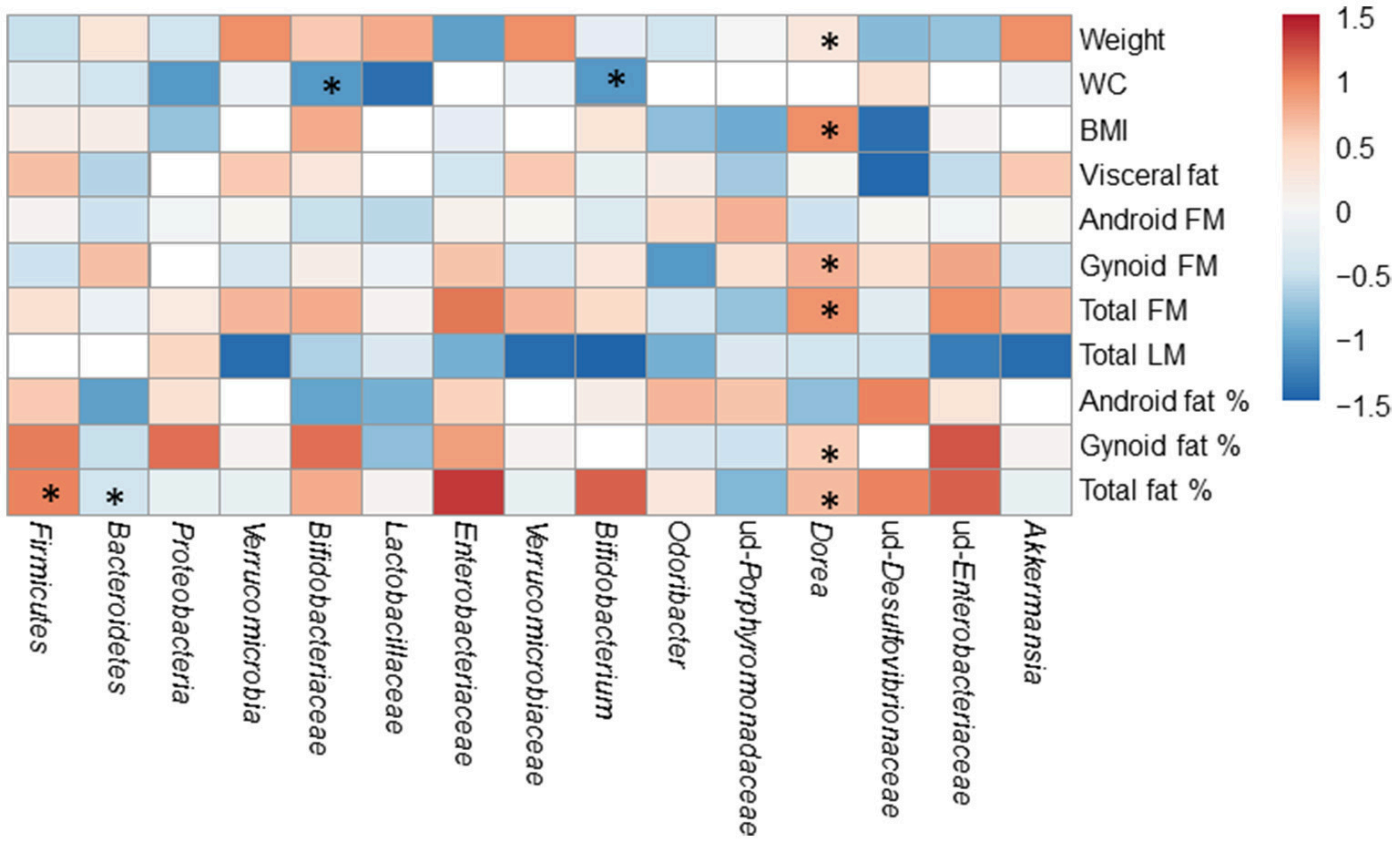
The associations of the gut microbiota with body composition before the exercise period. The significant associations are marked with asterisks (^*^). The numeric colored scale bar represents the Spearman correlation coefficients.

The associations of gut microbiota with body composition were studied before and after the exercise training. The significant associations of gut microbiota with body composition before the exercise period are displayed in Figure 6. After the exercise period, *Bifidobacterium* genus correlated with waist circumference (*r* = −0.580, *p* = 0.036), and *Dorea* with weight (*r* = 0.591, *p* = 0.033), android FM (*r* = 0.630, *p* = 0.021) and waist circumefrence (*r* = 0.674, *p* = 0.012).

## Discussion

In this study we showed that six-weeks endurance exercise intervention modestly modified the composition and functions of the gut microbiota without greatly affecting systemic metabolites or body composition. However, the exercise intervention was successful as the maximal power and maximum rate of oxygen consumption increased. Since no evident changes in weight, body composition and diet occurred during the exercise training, it can be proposed that the effects on gut microbiota were related to the exercise training. The only change in the diet after the exercise period was a slight increase in energy derived from starch. However, this has not likely affected the metagenome as starch is broken down into glucose moieties and metagenome analysis revealed no increases in glucose metabolism genes.

The major findings of this study indicate that the six-weeks guided endurance exercise training induced modest shifts in gut microbiota both at taxonomical and functional level. In accordance, animal studies by us and others have reported that high-intensity endurance training modified the gut microbiota (Campbell et al., 2016; Denou et al., 2016) and, that intrinsic aerobic capacity impacts the microbiota composition (Pekkala et al., 2017). In recent times, accumulating attention has been addressed to the possible link between gut microbiota and physical fitness also in humans. It seems that both compositional and functional differencies in the gut microbiota exist between professional athletes and their BMI-matched sedentary counterparts (Clarke et al., 2014) as well as between physically active and sedentary women (Bressa et al., 2017). But interestingly, contradictory findings from the human exercise interventions have been recently presented (Allen et al., 2017; Cronin et al., 2018). In contrast to our study, after eight-weeks of non-guided, combined aerobic and resistance exercise, Cronin et al. failed to detect significant changes in the gut microbiota composition or functions. However, including both genders in the study may have hindered some of the discoveries, as gender differences in the gut microbiota are known to influence, for instance, metabolic outcomes and immunity (Fransen et al., 2017). Therefore, our study is not straightforward comparable to the study published by Cronin et al. On the other hand, similar to our results, Allen and co-workers reported compositional and β-diversity changes of the microbiota in response to six-weeks endurance training that resembled the intervention of our study (Allen et al., 2017). The authors concluded that the shifts were dependent on the obesity status and were reversed after cessation of the training.

Inter-individual variation in the gut microbiota composition and gene content is known to be vast (Lozupone et al., 2012). Hence, also the responsiviness of an individual to certain lifestyle changes, such as dietary intervention or increased physical activity varies greatly (Korpela and de Vos, 2016; Korpela et al., 2016) creating a challenge in studies designed to modify the gut microbiota. Therefore, we studied the effects of exercise training on the microbiome in a study setting in which the subjects served as their own controls. However, even with such study set up no effects of the intervention on gut microbiota were observed with lower sequencing depth, and modest changes were found with deeper metagenomic analysis. It was evident from Bray Curtis dissimilarity analysis that the difficulty in finding more than modest changes is partly due to that approximately only half of the subjects' microbiomes actually responded to the exercise training. This suggests that among the microbiota there may be exercise responsive and non-responsive taxa that will be discussed in the following paragraph. A similar concept of the so-called responder microbes was also raised in a diet intervention that attempted to ameliorate Type 2 diabetes by improving short chain fatty acid (SCFA) production from fibers (Zhao et al., 2018a). The authors treated 27 subjects with a high-fiber diet and found that despite harboring SCFA metabolism genes only 15 out of 154 potential taxa responded to the diet.

It is noteworthy that the exercise training increased the abundance of *Verrucomirobia*, a phylum that contains only a few species, and further *Akkermansia*, one of the major representatives of *Verrucomicrobia*. *Akkermansia* is suggested to be health-beneficial for its capacity to improve lipid oxidation in diet-induced obese mice (Everard et al., 2013). In agreement with our findings, several line of evidence suggest that these taxa respond to exercise. First, athletes harbored more *Akkermansia* than sedentary controls (Clarke et al., 2014) as did the active women compared to inactive (Bressa et al., 2017). However, it should be noted that in both studies the active and sedentary group differed from each other in dietary intakes, especially fiber intake. This may have influenced the outcomes as fiber-rich diet seem to promote *Akkermansia* growth (Everard et al., 2014). In our study the change in *Akkermansia* occurred independent of weight, body compostion and food intake. Second, a 4-days cross-country, military ski-march increased *Verrucomicrobia* abundance (Karl et al., 2017) supporting further the emplacement of this phyla among the exercise-responsive taxa. *Akkermansia* has been found to be lower in several conditions, such as during obesity, diabetes (Cani and de Vos, 2017) and therefore, if future studies are able to show that the low abundance is major contributor to these coinditions and exercise increases the abundace, exercise would likely improve the diseases.

Our endurance exercise intervention decreased the abundance of *Proteobacteria* and an unidentified genus of *Enterobacteriaceae*. The change in *Proteobacteria* occurred independent of weight, body compostion and food intake. An increased abundance of *Enterobacteriaceae* has been reported in obesity (Scheepers et al., 2015) and non-alcoholic steatohepatosis (Zhu et al., 2013), suggesting that these species may be health-detrimental. In agreement with our findings, exercise decreased *Proteobacteria* in rats with high aerobic capacity (Liu et al., 2015). However, to our knowledge, no studies in humans have reported associations between *Proteobacteria* and exercise or physical fitness that would support the responsiveness of this taxon to exercise training. But *Dorea, Odoribacter* and a yet unidentified genus of *Desulfovibrionaceae* may be responders. Corroborating our findings *Odoribacter* and unidentified *Desulfovibrionaceae* were lower in active women than sedentary (Bressa et al., 2017) and in rats the latter decreased in response to voluntary exercise (Liu et al., 2015). In animal models exercise has boosted the abundance of *Dorea* (Allen et al., 2015; Denou et al., 2016) while in a human study it increased after cessation of the training and not in response to exercise (Allen et al., 2017). However, the changes in *Dorea, Odoribacter* and a yet unidentified genus of *Desulfovibrionaceae* were dependent on age, weight, body fat %, android fat %, intake of total energy, sucrose or fiber. We therefore propose that *Verrucomicrobia* and *Akkermansia* are true exercise-responsive taxa.

It was recently demonstrated by targeted gene amplification, not metagenomics, that in addition to the influence on the gut microbiota composition, exercise training increased the gene encoding butyrate forming Butyryl CoA: Acetate CoA transferase (Allen et al., 2017), which occured due to an increase in butyrate-producing bacteria. However, in our study, despite the favorable taxonomic shifts in butyrate-producing *Akkermansia* and *Dorea*, exercise training did not increase butyrate metabolism genes but reduced several metagenomic functions including those related to fructose, mannose, alanine and aromatic amino acids metabolism. Some of these functional changes may have simple biological explanations. As exercise is known to increase absorption of fructose (Fujisawa et al., 1993) that mainly occurs in the small intestine, it is possible that less sugars reach the colon where the majority of carbohydrate-metabolizing microbes reside (Krajmalnik-Brown et al., 2012). This could contribute to the reduction in fructose and mannose metabolism genes. Similarly, the statistically non-signifcant decline in protein ingestion during the exercise period can contribute to the decrease of metagenomic functions related to amino acids metabolism. Nevertheless, a prolonged protein supplementation did not affect the gut microbiome (Cronin et al., 2018) and therefore the real effect of reduced protein intake can be questioned. In summary, it seems that exercise training without an accompanying robust dietary change may not considerably affect the metagenomic functions. However, it should be noted that although food records are considered as a reliable dietary assessment tool, they are prone to under-reporting, and social-desirability and social approval bias, which could e.g., result in lower assessed energy intake, and over-reporting of healthy foods and underreporting of unhealthy foods. In agreement with no gross changes in the diet, the modest functional changes in gut microbiota were not translated into major systemic metabolic changes, as metabolomics analyses showed changes only in phospholipids and cholesterol in large VLDL particles in response to exercise. However, these changes are beneficial for cardiometabolic health as VLDL transports lipids from liver to peripheral tissues and thus has detrimental cardiovascular effects (Ren et al., 2010). The VLDL particle reducing effect of exercise is supported by our own study showing that long-term leisure time physical activity was associated with lower cholesterol in VLDL (Kujala et al., 2013).

Finally, exercise training decreased VAP-1 activity, which can have beneficial anti-inflammatory effects especially on vasculature, though the underlying mechanisms could not be determined in this study. Exercise training possibly produces a yet unknown endogenous inhibitor of VAP-1, but it remains to be determined how exercise regulates VAP-1 activity without affecting its protein concentration. Another immunologic change was the increased expression of *TLR5* in response to exercise training. Our finding is in agreement with the report showing that a prolonged bout of strenuous exercise increases the expression of TLRs (Gleeson, 2007). In addition, in mice, exhaustive exercise up-regulates flagellin-mediated inflammation by increasing *TLR5* expression (Uchida et al., 2014).

In conclusion, a successfully conducted six-weeks guided endurance exercise training modified the composition and functions of gut microbiota without greatly affecting systemic metabolites. These shifts were independent of gross changes in weight, body composition or diet. Based on our data and existing literature we propose that especially *Akkermansia* is an exercise-responsive taxon. Our results warrant the need for further studies in larger cohorts to determine whether all exercise types other than endurance exercise also modify the gut metagenome. In the future, it would be important to confirm the gut microbiota findings with longer/larger exercise trials and to see whether in long-term the exercise-induced gut microbiota changes lead to changes in the metabolome.

## Author contributions

SP, EM, UK, and JA designed the study. UK was study physician. SP, EM, JA, MH, AK, SJ, and GD performed the laboratory analyses. EM, SPi, LE, and AK analyzed the 16S data. SPi, LE, and GD deposited the sequence data. SP, PP, and GD analyzed the metagenome data. SP and JA analyzed the metabolomics data. KP analyzed dietary data. SP, EM, JA, SPi, LE, and KP did the statistical analyses. SP, SJ, and PH financed the experiments. SP, EM, KP, and JA wrote manuscript. All authors revised and commented on the manuscript and approved the final version.

### Conflict of interest statement

The authors declare that the research was conducted in the absence of any commercial or financial relationships that could be construed as a potential conflict of interest.

## References

[B1] Aalto K. Havulinna A. S. Jalkanen S. Salomaa V. Salmi M. (2014). Soluble vascular adhesion protein-1 predicts incident major adverse cardiovascular events and improves reclassification in a finnish prospective cohort study. Circ. Cardiovasc. Genet. 7, 529–535. 10.1161/CIRCGENETICS.113.000543 24850810

[B2] Aalto K. Maksimow M. Juonala M. Viikari J. Jula A. Kahonen M.. (2012). Soluble vascular adhesion protein-1 correlates with cardiovascular risk factors and early atherosclerotic manifestations. Arterioscler. Thromb. Vasc. Biol. 32, 523–532. 10.1161/ATVBAHA.111.238030 22116093

[B3] Allen J. M. Berg Miller M. E. Pence B. D. Whitlock K. Nehra V. Gaskins H. R.. (2015). Voluntary and forced exercise differentially alters the gut microbiome in C57BL/6J mice. J. Appl. Physiol. 118, 1059–1066. 10.1152/japplphysiol.01077.2014 25678701

[B4] Allen J. M. Mailing L. J. Niemiro G. M. Moore R. Cook M. D. White B. A.. (2017). Exercise alters gut microbiota composition and function in lean and obese humans. Med. Sci. Sports Exerc. 50, 747–757. 10.1249/MSS.0000000000001495 29166320

[B5] Aunola S. Rusko H. (1986). Aerobic and anaerobic thresholds determined from venous lactate or from ventilation and gas exchange in relation to muscle fiber composition. Int. J. Sports Med. 7, 161–166. 10.1055/s-2008-1025755 3733312

[B6] Borg G. (1970). Perceived exertion as an indicator of somatic stress. Scand. J. Rehabil. Med. 2, 92–98. 5523831

[B7] Bressa C. Bailen-Andrino M. Perez-Santiago J. Gonzalez-Soltero R. Perez M. Montalvo-Lominchar M. G.. (2017). Differences in gut microbiota profile between women with active lifestyle and sedentary women. PLoS ONE 12:e0171352. 10.1371/journal.pone.0171352 28187199 PMC5302835

[B8] Campbell S. C. Wisniewski P. J. Noji M. McGuinness L. R. Haggblom M. M. Lightfoot S. A.. (2016). The effect of diet and exercise on intestinal integrity and microbial diversity in mice. PLoS ONE 11:e0150502. 10.1371/journal.pone.0150502 26954359 PMC4783017

[B9] Cani P. D. Amar J. Iglesias M. A. Poggi M. Knauf C. Bastelica D.. (2007). Metabolic endotoxemia initiates obesity and insulin resistance. Diabetes 56, 1761–1772. 10.2337/db06-1491 17456850

[B10] Cani P. D. de Vos W. M. (2017). Next-generation beneficial microbes: the case of akkermansia muciniphila. Front. Microbiol. 8:1765. 10.3389/fmicb.2017.01765 29018410 PMC5614963

[B11] Clark A. Mach N. (2017). The crosstalk between the gut microbiota and mitochondria during exercise. Front. Physiol. 8:319. 10.3389/fphys.2017.00319 28579962 PMC5437217

[B12] Clarke S. F. Murphy E. F. O'Sullivan O. Lucey A. J. Humphreys M. Hogan A.. (2014). Exercise and associated dietary extremes impact on gut microbial diversity. Gut 63, 1913–1920. 10.1136/gutjnl-2013-306541 25021423

[B13] Cronin O. Barton W. Skuse P. Penney N. C. Garcia-Perez I. Murphy E. F.. (2018). A prospective metagenomic and metabolomic analysis of the impact of exercise and/or whey protein supplementation on the gut microbiome of sedentary adults. mSystems 3:e00044. 10.1128/mSystems.00044-18 29719871 PMC5915698

[B14] Denou E. Marcinko K. Surette M. G. Steinberg G. R. Schertzer J. D. (2016). High-intensity exercise training increases the diversity and metabolic capacity of the mouse distal gut microbiota during diet-induced obesity. Am. J. Physiol. Endocrinol. Metab. 310, E982–E993. 10.1152/ajpendo.00537.2015 27117007 PMC4935139

[B15] Everard A. Belzer C. Geurts L. Ouwerkerk J. P. Druart C. Bindels L. B.. (2013). Cross-talk between Akkermansia muciniphila and intestinal epithelium controls diet-induced obesity. Proc. Natl. Acad. Sci. U.S.A. 110, 9066–9071. 10.1073/pnas.1219451110 23671105 PMC3670398

[B16] Everard A. Lazarevic V. Gaia N. Johansson M. Stahlman M. Backhed F.. (2014). Microbiome of prebiotic-treated mice reveals novel targets involved in host response during obesity. ISME J. 8, 2116–2130. 10.1038/ismej.2014.45 24694712 PMC4163056

[B17] Fransen F. van Beek A. A. Borghuis T. Meijer B. Hugenholtz F. van der Gaast-de Jongh C.. (2017). The impact of gut microbiota on gender-specific differences in immunity. Front. Immunol. 8:754. 10.3389/fimmu.2017.00754 28713378 PMC5491612

[B18] Fujisawa T. Mulligan K. Wada L. Schumacher L. Riby J. Kretchmer N. (1993). The effect of exercise on fructose absorption. Am. J. Clin. Nutr. 58, 75–79. 8317393 10.1093/ajcn/58.1.75

[B19] Galperin M. Y. Makarova K. S. Wolf Y. I. Koonin E. V. (2015). Expanded microbial genome coverage and improved protein family annotation in the COG database. Nucleic Acids Res. 43, D261–D269. 10.1093/nar/gku1223 25428365 PMC4383993

[B20] Gleeson M. (2007). Immune function in sport and exercise. J. Appl. Physiol. 103, 693–699. 10.1152/japplphysiol.00008.2007 17303714

[B21] Karl J. P. Margolis L. M. Madslien E. H. Murphy N. E. Castellani J. W. Gundersen Y.. (2017). Changes in intestinal microbiota composition and metabolism coincide with increased intestinal permeability in young adults under prolonged physiological stress. Am. J. Physiol. Gastrointest. Liver Physiol. 312, G559–G571. 10.1152/ajpgi.00066.2017 28336545

[B22] Kettunen J. Demirkan A. Wurtz P. Draisma H. H. Haller T. Rawal R.. (2016). Genome-wide study for circulating metabolites identifies 62 loci and reveals novel systemic effects of LPA. Nat. Commun. 7:11122. 10.1038/ncomms11122 27005778 PMC4814583

[B23] Kopylova E. Noe L. Touzet H. (2012). SortMeRNA: fast and accurate filtering of ribosomal RNAs in metatranscriptomic data. Bioinformatics 28, 3211–3217. 10.1093/bioinformatics/bts611 23071270

[B24] Korpela K. de Vos W. M. (2016). Antibiotic use in childhood alters the gut microbiota and predisposes to overweight. Microb. Cell 3, 296–298. 10.15698/mic2016.07.514 28357367 PMC5354595

[B25] Korpela K. Salonen A. Virta L. J. Kekkonen R. A. Forslund K. Bork P.. (2016). Intestinal microbiome is related to lifetime antibiotic use in Finnish pre-school children. Nat. Commun. 7:10410. 10.1038/ncomms10410 26811868 PMC4737757

[B26] Krajmalnik-Brown R. Ilhan Z. E. Kang D. W. DiBaise J. K. (2012). Effects of gut microbes on nutrient absorption and energy regulation. Nutr. Clin. Pract. 27, 201–214. 10.1177/0884533611436116 22367888 PMC3601187

[B27] Kujala U. M. Makinen V. P. Heinonen I. Soininen P. Kangas A. J. Leskinen T. H.. (2013). Long-term leisure-time physical activity and serum metabolome. Circulation 127, 340–348. 10.1161/CIRCULATIONAHA.112.105551 23258601

[B28] Lambert J. E. Myslicki J. P. Bomhof M. R. Belke D. D. Shearer J. Reimer R. A. (2015). Exercise training modifies gut microbiota in normal and diabetic mice. Appl. Physiol. Nutr. Metab. 40, 749–752. 10.1139/apnm-2014-0452 25962839

[B29] Ley R. E. Turnbaugh P. J. Klein S. Gordon J. I. (2006). Microbial ecology: human gut microbes associated with obesity. Nature 444, 1022–1023. 10.1038/4441022a 17183309

[B30] Liu T. W. Park Y. M. Holscher H. D. Padilla J. Scroggins R. J. Welly R.. (2015). Physical activity differentially affects the cecal microbiota of ovariectomized female rats selectively bred for high and low aerobic capacity. PLoS ONE 10:e0136150. 10.1371/journal.pone.0136150 26301712 PMC4547806

[B31] Lozupone C. A. Stombaugh J. I. Gordon J. I. Jansson J. K. Knight R. (2012). Diversity, stability and resilience of the human gut microbiota. Nature 489, 220–230. 10.1038/nature11550 22972295 PMC3577372

[B32] McKenzie A. I. Briggs R. A. Barrows K. M. Nelson D. S. Kwon O. S. Hopkins P. N.. (2017). A pilot study examining the impact of exercise training on skeletal muscle genes related to the TLR signaling pathway in older adults following hip fracture recovery. J. Appl. Physiol. 122, 68–75. 10.1152/japplphysiol.00714.2016 27789770 PMC7002863

[B33] Moseley L. Jeukendrup A. E. (2001). The reliability of cycling efficiency. Med. Sci. Sports Exerc. 33, 621–627. 10.1097/00005768-200104000-00017 11283439

[B34] Munukka E. Pekkala S. Wiklund P. Rasool O. Borra R. Kong L.. (2014). Gut-adipose tissue axis in hepatic fat accumulation in humans. J. Hepatol. 61, 132–138. 10.1016/j.jhep.2014.02.020. 24613361

[B35] Munukka E. Wiklund P. Pekkala S. Volgyi E. Xu L. Cheng S.. (2012). Women with and without metabolic disorder differ in their gut microbiota composition. Obesity (Silver. Spring). 20, 1082–1087. 10.1038/oby.2012.8 22293842

[B36] Pekkala S. Lensu S. Nokia M. Vanhatalo S. Koch L. G. Britton S. L.. (2017). Intrinsic aerobic capacity governs the associations between gut microbiota composition and fat metabolism age-dependently in rat siblings. Physiol. Genomics 49, 733–746. 10.1152/physiolgenomics.00081.2017 29030493 PMC5814668

[B37] Pruesse E. Peplies J. Glockner F. O. (2012). SINA: accurate high-throughput multiple sequence alignment of ribosomal RNA genes. Bioinformatics 28, 1823–1829. 10.1093/bioinformatics/bts252 22556368 PMC3389763

[B38] Pruesse E. Quast C. Knittel K. Fuchs B. M. Ludwig W. Peplies J.. (2007). SILVA: a comprehensive online resource for quality checked and aligned ribosomal RNA sequence data compatible with ARB. Nucleic Acids Res. 35, 7188–7196. 10.1093/nar/gkm864 17947321 PMC2175337

[B39] Ren J. Grundy S. M. Liu J. Wang W. Wang M. Sun J.. (2010). Long-term coronary heart disease risk associated with very-low-density lipoprotein cholesterol in Chinese: the results of a 15-year Chinese Multi-Provincial Cohort Study (CMCS). Atherosclerosis 211, 327–332. 10.1016/j.atherosclerosis.2010.02.020 20223457

[B40] Rintala A. Riikonen I. Toivonen A. Pietila S. Munukka E. Pursiheimo J. P.. (2018). Early fecal microbiota composition in children who later develop celiac disease and associated autoimmunity. Scand. J. Gastroenterol. 53, 403–409. 10.1080/00365521.2018.1444788 29504486

[B41] Salmi M. Jalkanen S. (2017). Vascular adhesion protein-1: a cell surface amine oxidase in translation. Antioxid. Redox Signal. [Epub ahead of print]. 10.1089/ars.2017.7418 29065711 PMC6306676

[B42] Scheepers L. E. Penders J. Mbakwa C. A. Thijs C. Mommers M. Arts I. C. (2015). The intestinal microbiota composition and weight development in children: the KOALA Birth Cohort Study. Int. J. Obes. (Lond). 39, 16–25. 10.1038/ijo.2014.178 25298274

[B43] Schmieder R. Edwards R. (2011). Quality control and preprocessing of metagenomic datasets. Bioinformatics 27, 863–864. 10.1093/bioinformatics/btr026 21278185 PMC3051327

[B44] Sekirov I. Russell S. L. Antunes L. C. Finlay B. B. (2010). Gut microbiota in health and disease. Physiol. Rev. 90, 859–904. 10.1152/physrev.00045.2009 20664075

[B45] Shukla S. K. Cook D. Meyer J. Vernon S. D. Le T. Clevidence D.. (2015). Changes in gut and plasma microbiome following exercise challenge in myalgic encephalomyelitis/chronic fatigue syndrome (ME/CFS). PLoS ONE 10:e0145453. 10.1371/journal.pone.0145453 26683192 PMC4684203

[B46] Tanaka H. Monahan K. D. Seals D. R. (2001). Age-predicted maximal heart rate revisited. J. Am. Coll. Cardiol. 37, 153–156. 10.1016/S0735-1097(00)01054-8 11153730

[B47] Tatusov R. L. Koonin E. V. Lipman D. J. (1997). A genomic perspective on protein families. Science 278, 631–637. 9381173 10.1126/science.278.5338.631

[B48] Turnbaugh P. J. Hamady M. Yatsunenko T. Cantarel B. L. Duncan A. Ley R. E.. (2009). A core gut microbiome in obese and lean twins. Nature 457, 480–484. 10.1038/nature07540 19043404 PMC2677729

[B49] Uchida M. Oyanagi E. Kawanishi N. Iemitsu M. Miyachi M. Kremenik M. J.. (2014). Exhaustive exercise increases the TNF-alpha production in response to flagellin via the upregulation of toll-like receptor 5 in the large intestine in mice. Immunol. Lett. 158, 151–158. 10.1016/j.imlet.2013.12.021 24412598

[B50] Welly R. J. Liu T. W. Zidon T. M. Rowles J. L. III Park Y. M. Smith T. N.. (2016). Comparison of diet versus exercise on metabolic function and gut microbiota in obese rats. Med. Sci. Sports Exerc. 48, 1688–1698. 10.1249/MSS.0000000000000964 27128671 PMC4987217

[B51] Yu P. H. Lu L. X. Fan H. Kazachkov M. Jiang Z. J. Jalkanen S.. (2006). Involvement of semicarbazide-sensitive amine oxidase-mediated deamination in lipopolysaccharide-induced pulmonary inflammation. Am. J. Pathol. 168, 718–726. 10.2353/ajpath.2006.050970 16507887 PMC1606534

[B52] Zhao L. Zhang F. Ding X. Wu G. Lam Y. Y. Wang X.. (2018a). Gut bacteria selectively promoted by dietary fibers alleviate type 2 diabetes. Science 359, 1151–1156. 10.1126/science.aao5774 29590046

[B53] Zhao X. Zhang Z. Hu B. Huang W. Yuan C. Zou L. (2018b). Response of gut microbiota to metabolite changes induced by endurance exercise. Front. Microbiol. 9:765. 10.3389/fmicb.2018.00765 29731746 PMC5920010

[B54] Zhu L. Baker S. S. Gill C. Liu W. Alkhouri R. Baker R. D.. (2013). Characterization of gut microbiomes in nonalcoholic steatohepatitis (NASH) patients: a connection between endogenous alcohol and NASH. Hepatology 57, 601–609. 10.1002/hep.26093 23055155

